# A feline case of multiple myeloma treated with bortezomib

**DOI:** 10.1186/s12917-022-03484-1

**Published:** 2022-11-03

**Authors:** Hiroyuki Tani, Ryo Miyamoto, Teruki Miyazaki, Shingo Oniki, Kyoichi Tamura, Makoto Bonkobara

**Affiliations:** 1 Department of Veterinary Clinical Pathology, Nippon Veterinary and Life Science University, Tokyo, Japan; 2 Musashikoganei haru dog and cat hospital, Tokyo, Japan; 3 Research Center for Animal Life Science, Nippon Veterinary and Life Science University, Tokyo, Japan

**Keywords:** Cat, Multiple myeloma, Bortezomib

## Abstract

**Background:**

Multiple myeloma (MM) is an uncommon neoplasm in cats. There is no established standard of treatment due to the rare occurrence of this disease in cats. Bortezomib is a proteasome inhibitor that serves as the first-line drug for MM in humans, but its effectiveness currently is unknown in feline MM. We present here the case report of a feline MM that exhibited a favorable response to bortezomib.

**Case presentation:**

The case was an 11-year-old non-castrated male domestic cat with light-chain MM presenting with clinical symptoms (anorexia, fatigue, and vomiting), mild azotemia, and pancytopenia. The cat failed on melphalan with prednisolone (MP), so bortezomib (Velcade) was initiated on Day 88. A total of 6 cycles of the treatment was performed, with each treatment cycle consisting of twice-weekly subcutaneous administration for 2 weeks followed by a 1-week rest. The dose of bortezomib was 0.7 mg/m^2^ for first week and 1.0 mg/m^2^ for second week in the first cycle. A dose of 0.7 mg/m^2^ was used for subsequent cycles. Prednisolone was used concomitantly in the first 2 cycles. Following treatment with bortezomib, clinical symptoms disappeared and a decrease in serum globulin and recovery of pancytopenia were noted. A monoclonal gammopathy, overproduction of serum immunoglobulin light chain, and Bence-Jones proteinuria that existed at diagnosis were undetectable on Day 123. A monoclonal gammopathy also was not detectable at the end of the bortezomib treatment (Day 213). Anorexia, fatigue, and marked bone marrow toxicity were experienced when bortezomib was administrated at a dose of 1.0 mg/m^2^, while no recognizable toxicity was observed at a dose of 0.7 mg/m^2^ throughout the treatment period. The case was placed on follow-up and there was no evidence of relapse as of Day 243.

**Conclusions:**

Bortezomib was effective and durable for the treatment of this case of feline MM after failure with MP. Bortezomib was well-tolerated in this cat at a dose of 0.7 mg/m^2^, but not at 1.0 mg/m^2^. Bortezomib appears to be a drug worthy of further study for the treatment of feline MM.

**Supplementary Information:**

The online version contains supplementary material available at 10.1186/s12917-022-03484-1.

## Background

Multiple myeloma (MM) is an uncommon neoplasm in cats. Due to the uncommon occurrence of this disease in cats, there is no established standard of treatment for this species. Chemotherapy using melphalan combined with prednisolone/prednisone (MP), a treatment often used for the treatment of canine MM [1] and formerly used as a first-line therapy in human MM, has been reported in some feline MM cases [2, 3]. In those reports, favorable responses to such therapy were noted; three out of eight patients had a survival more than 6 months [2] and one out of seven patients had achieved survival of 6 months [3]. However, there were cases that did not respond or responded but had survival of several weeks or less.

A proteasome inhibitor, bortezomib, currently serves as the first-line drug for treatment of MM in humans. This compound is a breakthrough drug for human MM that previously had been treated with MP as a first-line treatment for an extended period. Bortezomib reversibly binds to the 26S proteasome, preventing degradation of various pro-apoptotic factors by the proteasome and resulting in activation of programmed cell death in neoplastic cells which generates the therapeutic effects in human patients with MM [4]. Although bortezomib nowadays is widely used for treatment of MM in humans, the therapeutic utility of this compound in feline MM currently is unknown.

Here, we report a feline case of MM that responded well to bortezomib after treatment failure with MP.

## Case presentation

**Fig. 1:**
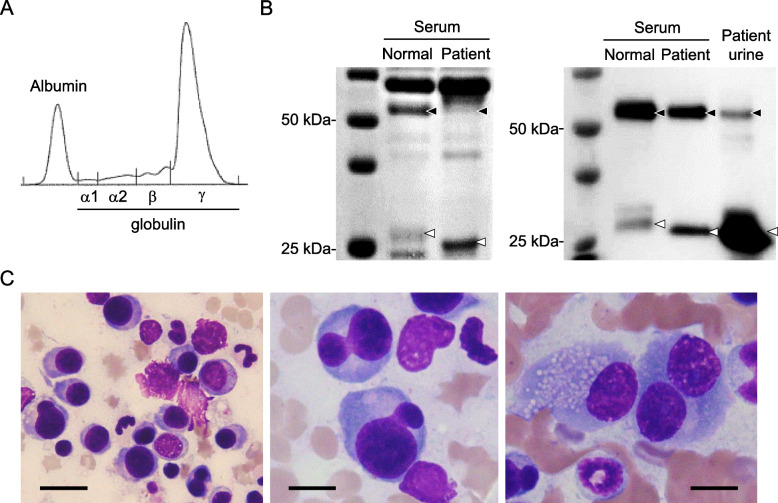
Diagnostic findings of multiple myeloma in the cat. **A** Monoclonal gammopathy as assessed by agarose gel serum protein electrophoresis. **B** Left panel: serum protein analysis by sodium dodecyl sulphate-polyacrylamide gel electrophoresis with Coomassie brilliant blue staining. Bands at ~ 59 kDa (the size of the immunoglobulin heavy chain) and ~ 27 kDa (the size of the immunoglobulin light chain) are indicated by black and white arrowheads, respectively. Specifically, in contrast to normal cat serum, serum from the cat with multiple myeloma provided a more intense band at ~ 27 kDa, and a fainter band at ~ 59 kDa. Right panel: western blotting analysis of serum (normal and patient) and patient urine using anti-feline gamma immunoglobulin (heavy and light chain) antibody. Immunoreactive bands were detected at ~ 59 kDa (black arrowhead; immunoglobulin heavy chain) and ~ 27 kDa (white arrowhead; immunoglobulin light chain) in sera from both normal and patient, confirming that the bands in the left panel (~ 59 kDa and ~ 27 kDa) were immunoglobulin heavy chain and light chain, respectively. An immunoreactive band at ~ 27 kDa (white arrowhead, Bence-Jones protein) and a weak band at ~ 59 kDa (black arrowhead, immunoglobulin heavy chain) were detected in patient urine. **C** Bone marrow smear stained with Wright-Giemsa (400x in left panel and 1000x in middle and right panels). Atypical plasma cell infiltration in the bone marrow was observed. Left panel: anisocytosis and anisokaryosis of plasma cells; middle panel: plasma cells with bizarrely shaped nucleus; right panel: binucleated plasma cell, plasma cells with visible nucleoli, and plasma cell with Russell body-like inclusions. Bars: left panel, 20 μm; middle and right panels, 10 μm. Equipment: microscope, Olympus BX50; objective lenses, Olympus UPlanFl 40x/0.75 and UPlanFl 100x/1.30 oil; image acquisition system, Olympus DP12

The case was an 11-year-old non-castrated male domestic cat that presented with a two-week history of anorexia, fatigue, and vomiting. The cat exhibited pancytopenia, characterized by nonregenerative anemia (hematocrit, 22% [reference range, 30.3–52.3%]; red blood cell count, 4.33 × 10^6^/μL [reference range, 6.54–12.2 × 10^6^/μL]; hemoglobin, 7.3 g/dL [reference range, 9.8–16.2 g/dL]; reticulocyte count, 3.9 × 10^3^/μL), leukopenia (2750/μL; reference range, 2870-17,020/μL) with neutropenia (1320/μL, reference range 2300-10,290/μL), and thrombocytopenia (39 × 10^3^/μL; reference range, 151–600 × 10^3^/μL). Serum chemistry revealed azotemia (blood urea nitrogen, 35.1 mg/dL [reference range, 17.6–32.8 mg/dL]; creatinine, 2.5 mg/dL [reference range 0.9–2.1 mg/dL]) with proteinuria. No abnormality was noted in the liver and electrolyte panels. The cat exhibited hyperproteinemia (10.9 g/dL; reference range, 5.7–7.8 g/dL) and hyperglobulinemia (7.5 g/dL; reference range, 2.7–5.2 g/dL) with a monoclonal gammopathy by agarose gel serum protein electrophoresis test (Fig. 1A). Serum protein analysis by sodium dodecyl sulphate-polyacrylamide gel electrophoresis (SDS-PAGE) with Coomassie brilliant blue staining (Fig. 1B, left panel) and western blotting (Fig. 1B, right panel) revealed overproduction of immunoglobulin light chain. The presence of immunoglobulin light chain in urine (Bence-Jones proteinuria) along with some immunoglobulin heavy chain (which may indicate the presence of glomerulopathy due to light chain nephrotoxicity) was detected by the same western blot (Fig. 1B, right panel). Details of the SDS-PAGE with Coomassie brilliant blue staining and western blotting are provided in Additional file 1 (original images of Fig. 1B are provided in Additional file 2). No abnormality was noted on thoracic and abdominal radiographs or on abdominal ultrasound scan. Bone marrow aspiration biopsy revealed bone marrow plasmacytosis (> 20% of bone marrow cells), in which the plasma cells were scattered or formed focal clusters. The plasma cells exhibited anisocytosis and anisokaryosis (Fig. 1C, left panel), and some had a bizarrely shaped nuclei (Fig. 1C, middle panel). Binucleated plasma cells, plasma cells with visible single or multiple nucleoli, and plasma cells with Russell body-like inclusions were observed occasionally (Fig. 1C, right panel). Monoclonality in immunoglobulin lambda light chain was detected by PCR clonality testing using the bone marrow aspirate. Based on the findings including a monoclonal gammopathy caused by overproduction of immunoglobulin light chain, Bence-Jones proteinuria, and a monoclonal neoplastic plasma cell expansion in bone marrow, this case was diagnosed as MM, specifically a variant light-chain MM. The day of diagnosis was defined as Day 1.

**Fig. 2:**
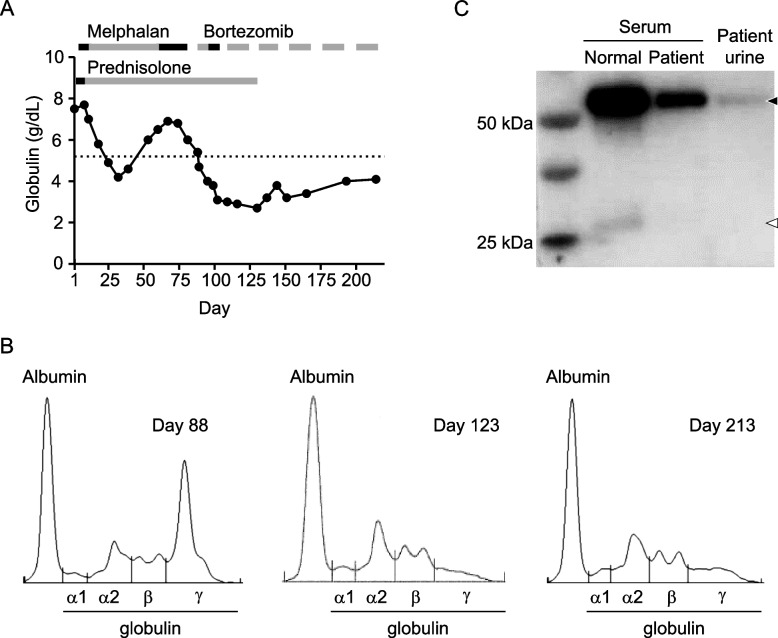
Response to treatment in the cat patient. **A** Treatment timeline and changes of serum globulin levels. Melphalan: black bar, 0.1 mg/kg, once daily; gray bar, 0.1 mg/kg, every other day. Bortezomib: black bar, 1.0 mg/m^2^, twice weekly; gray bar, 0.7 mg/m^2^, twice weekly; line gap, rest period. Prednisolone: black bar, 2 mg/kg; gray bar, 1 mg/kg. Graph shows changes of serum globulin levels. A dotted line in the graph indicates upper limit of the reference range of serum globulin in the cat (5.4 g/dL). **B** Results of agarose gel serum protein electrophoresis tests performed on Day 88, Day 123, and Day 213. **C** Western blotting analysis of serum (normal and patient) and patient urine using anti-feline gamma immunoglobulin (heavy and light chain) antibody. Black and white arrowheads indicate the sizes of band corresponding to the immunoglobulin heavy chain (~59 kDa) and light chain (~27 kDa), respectively

The cat underwent treatment with MP (Fig. 2A), which consisted of melphalan (0.1 mg/kg, orally, once daily [Day 4–10 and Day 60–81] or every other day [Day 11–59]; initiated on Day 4) combined with prednisolone (2 mg/kg with taper to 1 mg/kg, orally, once daily; initiated on Day 1). Although some responses to the treatment were observed, including decrease of serum globulin (Fig. 2A) and improvement of clinical symptoms at approximately Day 30, these responses were not durable and melphalan was discontinued on Day 81.

On Day 88, treatment of the cat with bortezomib (Velcade, Janssen Pharmaceutical K.K., Tokyo, Japan) was initiated (Fig. 2A). The treatment cycle consisted of subcutaneous administration of bortezomib (0.7 or 1.0 mg/m^2^) twice weekly for 2 weeks followed by a 1-week rest. Prednisolone (1 mg/kg, orally, once daily) was used concomitantly during the first two cycles. Complete blood cell counts (CBC) and serum chemistry tests were performed before each bortezomib dose administration in first three cycles and before the first bortezomib dose administration of a given week in the subsequent cycles. Additional CBC and serum chemistry tests and imaging tests (radiograph and/or ultrasound scan) were performed as needed during the treatment interval. Toxicities were graded according to the Veterinary Co-operative Oncology Group-Common Terminology Criteria for Adverse Events version 2 [5]. At the start of the bortezomib dosing interval, the cat exhibited moderate-to-severe clinical symptoms (anorexia and fatigue) and non-regenerative anemia (hematocrit, 26.8%; red blood cell count, 4.81 × 10^6^/μL; hemoglobin, 8.4 g/dL; reticulocyte count, 1.9 × 10^3^/μL) and leukopenia (1580/μL) with neutropenia (1320/μL). The thrombocyte count was near the lower limit of the reference range (157 × 10^3^/μL). The serum chemistry analysis revealed that the liver, renal, and electrolyte panels were within the respective reference range, although hyperproteinemia (8.8 g/dL) was observed. Serum globulin (5.4 g/dL) was slightly above the upper limit of the reference range but a monoclonal gammopathy was clearly present (Fig. 2B, left panel).

In the first cycle of bortezomib dosing, the compound was administered at 0.7 mg/m^2^ for the first week. Favorable responses including disappearance of clinical symptoms, restoration of neutrophil count to the normal range (4800/μL), and decrease of serum globulin (4.2 g/dL) (Fig. 2A), were observed at the end of the first week. The dose of bortezomib then was escalated to 1.0 mg/m^2^ in the second week (administered on Day 94 and Day 98). The cat subsequently exhibited grade 2 anorexia and grade 2 fatigue with grade 4 neutropenia (140/μL, non-febrile) and grade 3 thrombocytopenia (34 × 10^3^/μL) on the day after the second 1.0-mg/m^2^ dose. The cat then underwent treatment with antibiotics and supportive care. After a 1-week rest period, the clinical symptoms disappeared and neutrophil and thrombocyte counts were improved (6300/μL and 196 × 10^3^/μL, respectively). The dose of bortezomib therefore was set at 0.7 mg/m^2^ for subsequent cycles. The cat underwent a total of six cycles of bortezomib treatment (Day 88–213). During the second to sixth cycles of the treatment, the cat maintained in good condition and serum globulin values were consistently below the upper limit of the reference range (Fig. 2A). No neutropenia, thrombocytopenia, gastrointestinal symptoms, proteinuria, or abnormalities in serum chemistry were noted during this period, except that blood urea nitrogen and creatinine sometimes were slightly above the upper limit of their respective reference ranges. A monoclonal gammopathy, serum immunoglobulin light chain, and Bence-Jones proteinuria were not detectable on Day 123 (Fig. 2B, middle panel and Fig. 2C [an original image of Fig. 2C is provided in Additional file 2.]). A monoclonal gammopathy remained undetectable on Day 213 (Fig. 2B, right panel). Both hematocrit and red blood cell counts increased to normal ranges on Day 193 and remained within the normal range until the end of the bortezomib treatment interval. Because the reported median number of treatment cycles in human MM was 6 cycles [6] and this cat was in good condition at the end of 6 cycles of treatment, bortezomib treatment was stopped after 6 cycles. Following treatment, the case was placed on follow-up consisting of physical examination and blood tests every 2 weeks. The cat has remained in good condition and has exhibited no evidence of relapse as of Day 243.

## Discussion and conclusions

This work represents the first case report (to our knowledge) of feline MM successfully treated with bortezomib. Treatment with MP may be effective for feline cases of MM, as has been shown in previous reports [2, 3] and in the present case. However, no recommended therapeutic drug is known for feline MM after failure with MP, although use of cyclophosphamide and corticoids could be beneficial because the combination of these drugs has been demonstrated to have clinical activity in cats with myeloma-related disorder [7]. Given that the favorable response to bortezomib was noted in the present case following MP failure and the fact that bortezomib has therapeutic activity in human patients with MM who have relapsed after frontline therapy including MP [8], bortezomib may have potential for use as a salvage drug in feline MM after failure with MP. Moreover, in human patients with previously untreated MM, single-agent bortezomib therapy has been shown to be effective [9], and bortezomib plus MP has been shown to provide superior results compared to MP alone [10]. Considering these reports and the fact that bortezomib is currently used as the first-line drug for MM in humans, bortezomib also could have potential as a first-line single-agent or as an agent for use in combination with MP for the treatment of feline MM, although this will need to be investigated in further research.

Although little is known about behavior of MM in cats, a study of nine cats with MM [2] suggested that anemia, hypercalcemia, pathologic fractures, Bence-Jones proteinuria, azotemia, and persistent elevation of serum protein at 8 weeks after treatment and little/no clinical improvement were negative prognostic factors and reflected a more aggressive form of MM. In that study, survival time of the four cats with aggressive MM did not exceed 14 days (median, 5 days), while five cats with less-aggressive MM had a median survival of 387 days (range of 120 to 720 days). In human MM, anisocytosis and atypical morphology of neoplastic plasma cells, pancytopenia, and exhibiting light-chain MM have been shown to associate with shorter survival [11–13]. In the present report, the presenting case had pancytopenia, Bence-Jones proteinuria, and azotemia and exhibited only transient responses to MP. Additionally, this case was a light-chain MM and the neoplastic plasma cells exhibited anisocytosis with atypical morphology. Considering these points, MM in this case appeared to be aggressive form of MM; bortezomib seemed to contribute to the relatively long survival in this case.

For the treatment of the cat with bortezomib, prednisolone was concomitantly used in the first two cycles. Although anti-neoplastic effects of prednisolone in feline MM are not known, this agent may have provided potential benefit for this disease, so contributing to the observed tumor response in our study. However, given that the tumor recurred under MP treatment but remained well controlled more than 100 days after termination of prednisolone treatment, we infer that bortezomib likely played a pivotal role for the tumor response in this cat.

The standard dose of bortezomib in humans is 1.3 mg/m^2^. It has also been used with a reduced dose (1.0 mg/m^2^) or a minimal dose (0.7 mg/m^2^) in a phase 2 study of bortezomib in human MM when severe toxic effects were observed in patients with a standard dose [14]. Because the toxicity of bortezomib in cats was unknown, it was started at the human minimal dose of 0.7 mg/m^2^ and escalated to 1.0 mg/m^2^ in this study. Bortezomib, at a dose of 0.7 mg/m^2^, was well tolerated in this case, given that no recognizable toxic effect was observed at this dose level throughout the treatment period. Although mild azotemia sometimes was noted during the bortezomib treatment period, this symptom pre-existed in this case and was not worsened by bortezomib. At the same time, transient but marked bone marrow toxicity with clinical symptoms (anorexia and fatigue) were noted in this cat with dosing of bortezomib at 1.0 mg/m^2^. Notably, this dose was lower than the standard dose (1.3 mg/m^2^) used in humans. Although the maximum tolerated dose (MTD) of bortezomib is unknown in cats, the MTD has been shown to vary among species, with intravenous single-dose MTDs reported as 3.0 mg/m^2^ in the mice; 0.6 mg/m^2^ in the rat, 3.6 mg/m^2^ in the dog, and 1.2 mg/m^2^ in the monkey [15]. Thus, cat may be a species with higher susceptibility to bortezomib compared to humans. Alternatively, renal impairment in this cat may have led to the observed toxic effects of bortezomib at 1.0 mg/m^2^, given that bortezomib has been shown to cause more frequent and severe adverse events in clinical patients with renal failure in human MM [16].

In conclusion, bortezomib was effective and durable in the treatment of a case of feline MM after failure with MP. Bortezomib was well-tolerated in this cat at a dose of 0.7 mg/m^2^, but not at 1.0 mg/m^2^, with the higher dose being associated with anorexia, fatigue, and bone marrow toxicity. Further investigations on the toxicity and effectiveness of bortezomib for feline MM are encouraged.

## Supplementary Information

**Additional file 1.**

**Additional file 2.**

## Data Availability

The datasets used and/or analyzed during the current study are available from the corresponding author by reasonable request.

## Abbreviations

MM: Multiple myeloma
MP: Melphalan combined with prednisolone/prednisone
SDS-PAGE: Sodium dodecyl sulphate-polyacrylamide gel electrophoresis
