# Supplementary material for: A feline case of multiple myeloma treated with bortezomib
Source: BMC Vet Res. 2022 Nov 3;18:384. doi: 10.1186/s12917-022-03484-1 (PMC9632122; doi:10.1186/s12917-022-03484-1)
Supplement: Supplementary file 1 — Additional file 1. [file 12917_2022_3484_MOESM1_ESM.docx]

**Additional file 1**

**Sodium dodecyl sulphate-polyacrylamide gel electrophoresis with Coomassie brilliant blue staining**

Sera from the patient and a normal cat were diluted 400-fold with phosphate-buffered saline. Serum proteins were separated by 12% sodium dodecyl sulphate-polyacrylamide gel electrophoresis (SDS-PAGE) (3 μL serum sample/lane). The SDS-PAGE gels were stained with Coomassie brilliant blue and proteins were detected with a LAS-4000 (Fujifilm, Tokyo, Japan).

**Western blotting**

For western blotting, serum samples were prepared from the patient and a normal cat as described above; urine samples of the patient cat were concentrated (10-fold) using a Urine Protein Isolation and Concentration Kit (Funakoshi, Tokyo, Japan). Samples were subjected to 12% SDS-PAGE (3 μL for each serum or urine sample/lane). Proteins separated by SDS-PAGE were transferred to a polyvinylidene difluoride membrane (Bio-Rad, Hercules, CA) using a Trans-Blot Turbo Blotting System (Bio-Rad). After blocking of non-specific protein binding with 5% non-fat dry milk, the membrane was incubated with horseradish peroxidase-conjugated goat anti-feline gamma immunoglobulin heavy and light chains polyclonal antibody (Catalog No. A18757, Lot No. 39-79-101613; Invitrogen, Waltham, MA, USA). Immunoreactive bands were visualized using an enhanced chemiluminescence system (GE Healthcare, Chicago, IL, USA) and a LAS-4000.
