# Supplementary figures and images for: A feline case of multiple myeloma treated with bortezomib
Source: BMC Vet Res. 2022 Nov 3;18:384. doi: 10.1186/s12917-022-03484-1 (PMC9632122; doi:10.1186/s12917-022-03484-1)

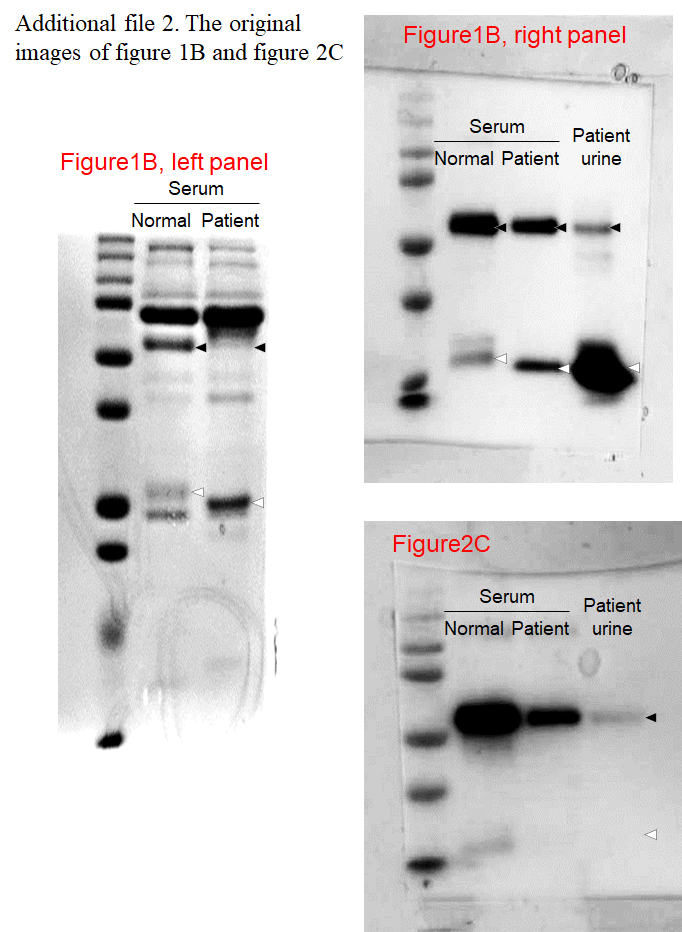

Supplement: Supplementary file 2 — Additional file 2. [file 12917_2022_3484_MOESM2_ESM.png]
